# Starch Food, Its Use and Abuse*Read before the Southern Kentucky Medical Association at Bowling Green November 3-4, 1897.

**Published:** 1898-03

**Authors:** D. G. Simmons


### STARCH FOOD, ITS USE AND ABUSE.*

BY D. G. SIMMONS, M. D.

There seems recently to have arisen a popular clamor against the use of starchy substances as food; so much so, indeed, that some have gone so far as to urge the abolition of bread, the principal source from which starch as a food is obtained, entirely from our tables. I suppose, however, this is only the extreme oscillation of the pendulum with the overzealous since special attention has been directed to starch food and the part its imperfect digestion plays in the production of disease.

In the brief consideration of the subject contemplated in this paper, it will be my purpose to refer, first, to the use of starch as a food and the importance of its use in the animal economy; second, to its abuse, and how such abuse is brought about; and third, the best means to correct its evil effects when injudiciously used.

First, as to the food value of starch. It has been said that the whole grain of the wheat cereal contains about every thing necessary for the repair of the physical man when properly prepared and prudently partaken of. Not only man, but nearly all the lower order of animals find a most valuable and a favorite food in the various cereals and other vegetable products containing amyloid and albuminoid elements in their varying proportions. So it is with all kinds of birds, and even fish are fond of bread crumbs and forms of food obtained from plant seed.

But it is only the amylaceous portion of our food that claims our attention at present. This is very much the larger portion of the grain of all cereals. The wonderful wisdom displayed in the general use of starch by the animal creation is attested by the purpose it subserves in the animal economy—a purpose absolutely necessary to the maintenance of body heat, and consequently to the maintenance of life.

In order that the starch may be appropriated to the wants of the system, it is necessary that it should pass through various processes and be transformed into various other substances. To successfully accomplish its purposes, it is necessary that it be well cooked, well masticated and thoroughly mixed with the saliva before its introduction into the stomach. The paramount importance of its being thoroughly mixed with the saliva should not be passed over without something more than a passing notice. Saliva, with its ptyalin, is the principal ferment and digestive agent of starchy matters, just as gastric juice, with its pepsin, is the principal instrument in the solution and digestion of albuminoids. If the starch should not obtain a sufficient admixture with saliva while in

*Read before the Southern Kentucky Medical Association at Bowling Green, November 3-4, 1897.

the mouth to effect its digestion, as it fails to get any aid from the stomach juices, it is likely, while contained in that organ, to be subjected to an abnormal form of fermentation with the evolution of gas, and become so decomposed that it becomes entirely unfit for the purpose it was intended to subserve, and it thus becomes an irritant to the stomach and bowels, as will be referred to and demonstrated further on.

Having then been thoroughly masticated and insalivated, it passes into the stomach, and before the acid of the gastric juice has had time to be secreted in sufficient quantity to neutralize the alkaline saliva, the starch has been sufficiently digested to pass along with the other food out of the stomach, which has been merely a side station for it while on its way to the liver.

The pancreatic juice completes its intestinal digestion, converting it into glucose, and it is taken up by the veins and carried to the liver, where it is again transformed into glycogen and stored up for future use. When it is drawn upon for use in the system, it is again changed into lactic acid, which is the form necessary for its combustion in the various tissues of the body, and thus it is appropriated to the maintenance of the body heat. So much for its use and purpose, and for the manner in which it is appropriated to effect that purpose.

We will now pass to the second section of the subject, viz., the abuse of this most valuable substance. An agent so potent for good is usually equally potent for evil if misused or abused. I presume it will be readily conceded that most of the dyspepsias, gastric catarrhs and other stomach disorders are attributable to the imperfect digestion of starch, coupled with eating too much. The main purpose of this paper is to endeavor to show how these dire complaints are the legitimate and almost inevitable result of a failure to properly digest starch.

We live in a bustling, hurrying, restless age, and especially in that character of country. We are taught, or at least encouraged, by precept and example from our infancy up, to complete the eating act in as short a time as possible—to bolt our food unmasticated, it being washed down by large draughts of fluid. Our railway eating-houses seem to have set the pace for most of our family tables. The starch thus utterly fails to get its due proportion of saliva, which is absolutely necessary for its healthy digestion, while the albuminoids in the form of lean meats, eggs, cheese and the glutinous portion of bread are unmasticated, and this throws double duty on the stomach. The stomach, partly by reason of this double duty and partly from excessive indulgence in table comforts, finally becomes exhausted and fails to honor the drafts made upon it in the digestion of the albuminoids, while the undigested starch is decomposed into irritable and corrosive acids and large volumes of sour gas, by reason of the warmth and moisture of the stomach, and the sugar taken more or less with each meal.

This gas in its turn, in the process of time, distends the stomach walls like a blown-up bladder, presses it up against the diaphragm and heart, producing pains, smothering, palpitations, intermission and all sorts of functional disturbances of that very sensitive and sympathetic organ. These acrid corrosive acids generated by decomposing starch, coupled with distension of the stomach by gases and overeating, result in chronic catarrhal inflammation of that organ, producing a glairy, tenacious mucus which mechanically coats and covers over the food taken into the stomach, and thus defeats the action of the gastric fluid on the albuminoids. In this condition the stomach is rarely empty of food, and never clear of this ropy, catarrhal mucus. The abundant evolution of gas incident to this state of things gradually dilates the stomach walls till frequently the organ occupies pretty much all the epigastric, left hypochondriac and left infra-axillary regions, thus compressing the lower and middle lobes of the left lung and displacing the heart from its normal position. This condition of gastrectasia, developed in greater or less degree, exists in an alarming number of cases, and I believe I am safe in attributing nine-tenths of them to the imperfect digestion of starch.

Since sugar has become so cheap, it is lavishly used by all classes in the different forms of toothsome dishes, and when these are coupled, as they generally are, with imperfectly digested amyloid substances, we may almost tremble for the future, for these stomach disturbances are growing more frequent daily, and are likely to continue if a better state of hygiene is not established.

How can these diseases be prevented, and how can they be successfully treated after they are developed? To prevent them we want to reform our habits of eating; we want to encourage the habit of spending more time at our tables; we want to eat more deliberately; we want to do more chewing of our solid foods, and thus soften them with the saliva in place of softening and washing them down prematurely with other fluids. In place of abolishing the use of bread in order to avoid the abuse of starch, we want to use bread and other starchy articles of diet judiciously and prudently, so as to obtain all their good effects and none of their evil effects. We want to masticate our solid food without the softening aid of any other fluids than those secreted by the mouth, and drink our fluids between swallows and alone. We want to learn to regulate the quantity eaten to the requirements of the system. We want to eat plain, wholesome food at regular hours, and avoid the habitual use of rich pastries and desserts. I believe I am safe in the assertion that fifty per cent. of our diseases, in this country at least, are the result, directly or indirectly, of imprudence in diet.

To use an illustration of Dr. Dio Lewis on another subject, some of these days when I get able I want to write a book. In order that its importance may be impressed properly upon its readers, I would like to sell the first edition of half a million for twenty-five dollars each, only I know that I could not find sale for them at any price, so I will distribute them gratuitously and promiscuously among the people. This book shall have but six pages, and each page shall have but one word, and that word shall be printed in great big circus type. These six words shall be, "No fluids in mouth with solids." Not only should all fluids be eschewed along with solids, but even juicy fruits and vegetables, which contain large proportions of water, should also be eaten alone. The matter stands thus: If dry solids are eaten alone it is impossible to swallow them till they have been properly softened by mastication and and insalivation; on the other hand, if any fluids are taken in the mouth with the solids, the latter are immediately softened thereby, and down goes the whole bolus, only half masticated and wholly unprovided with the saliva necessary to digest the starch, the stomach having to bear the burden, and after a while a generally disordered state of all the functions is the penalty.

Within the last few years I have seen a great number of cases of gastrectasia and gastric catarrh, ranging in age from little past infancy to old age, and in almost every case I have traced the origin of the diseases to eating too hurriedly, washing the food down unmasticated, and to undue indulgence in sweets. They are most frequently seen in those of sedentary lives, with a sluggish state of bowels, though all classes are subject to them under circumstances favorable for their development. Two cases were vigorous young farmers who, when them found themselves suffering and growing worse, rushed off to our osteopathic friends at Franklin, and after undergoing their routine of replacing a dislocated backbone and the usual pounding and punching over the stomach and bowels, came home at death's door, reporting that they grew worse every day while there. I am glad to say that under a proper observance of hygienic laws, and just enough medicine to give the stomach a boost for a few weeks, they made good recoveries.

The diseases incident to the non-conversion of starch, when not complicated by malignant disease, are usually susceptible to remedy, though they frequently tax the patience of both doctor and victim. The first consideration, of course, must be to remove the cause by requiring a rigid adherence to dietetic directions. Sweets must be entirely interdicted, so all desserts and pastries. All starchy substances must be used with great moderation, so that what is used may be thoroughly digested. Potatoes must be prohibited, both sweet and Irish. Toasted bread and Graham flour bread may be used moderately, as well as eggs, cheese, beef steak, roast lamb, fowls, oysters (fresh), most vegetables, milk, tea, and hot water before breakfast. The patient must be made to realize the importance of eating slowly, thoroughly masticating his food, and avoid the use of fluids during the chewing of solids.

These cases are always improved by having the ropy mucus washed out of the stomach with a syphon-tube and a warm alkaline antiseptic solution two or three times a week just previous to the breakfast hour. This is a decidedly unpleasant ordeal, and some patients utterly rebel against it.

In most cases of malconversion of starch there is usually associated, sooner or later, a catarrhal state of the gastric mucous membrane and impaired digestion of albuminoids; consequently, a rational treatment of these disorders should have reference to both disturbances; that is, the patient should be supplied with an artificial ptyalin for the conversion of starch into sugar and an artificial gastric juice to convert proteids into peptones. These artificial aids to digestion should not be used with the view of supplanting the action of the natural ferments, but only to supplement their action in their disordered condition until the natural processes can be restored.

As an aid for the conversion of starch the profession has come into possession of an invaluable remedy in Taka-Diastase. This substance has developed almost marvelous results along this line in the hands of all who have used it in properly selected cases, and it has evidently come to stay. I have used it with children as well as with adults, and thus far I have not been disappointed in it. The dose is small—one or two grains for an adult—it is readily soluble in water, and has no unpleasant taste. Parke, Davis & Co. have the manufacture of it. This agent should be given either during or immediately after meals, so as to complete the conversion of starch into maltose before the process is interrupted by the acid of the gastric juice. It acts very promptly in its converting power, and it resists the effects of the gastric acid much longer than does saliva. It also seems to exert considerable influence in the digestion of albuminoids.

For supp'ementary aid to the conversion of proteids, pure scale pepsin, given in doses of about three grains one hour after meals, seems to give better results than any thing I have used.

These medicines, together with proper attention to the bowels, a proper restriction of the diet, the avoidance for awhile of starchy and sweet articles of diet, the observance of the laws of hygiene in chewing solids alone, and the necessary outdoor exercise, will so improve the digestion and assimilation within a few weeks that nature will be able to complete the cure. If the medicines are indulged in too long, they exercise a weakening influence on the digestive organs.

I have frequently seen these greatly distened stomachs under this management gradually contract to their normal size and healthful tone within a few months, with a return to normal flesh and strength. Without these dietetic observances, all sorts of anti-dyspeptic remedies are almost valueless.—*American Practitioner and News.*

